# *Mycoplasma synoviae* induce spleen tissue damage and inflammatory response of chicken through oxidative stress and apoptosis

**DOI:** 10.1080/21505594.2023.2283895

**Published:** 2025-09-16

**Authors:** Lemiao Zhong, Chunlin Wu, Lvyan Liao, Yijian Wu

**Affiliations:** a University Key Laboratory for Integrated ChineseTraditional and Western Veterinary Medicine and Animal Healthcare, Fuzhou, Fujian Province, China; b Fujian Key Laboratory of Traditional Chinese Veterinary Medicine and Animal Health, Fujian Agriculture And Forestry University, Fuzhou, Fujian Province, China

**Keywords:** *Mycoplasma synovium*, spleen, oxidative stress, apoptosis, inflammation

## Abstract

*Mycoplasma synovium* (MS) is a prominent avian pathogen known to elicit robust inflammatory responses in birds while evading immune detection, often leading to chronic infection and immune compromise. The mechanisms underpinning MS-mediated splenic tissue damage in chickens, however, remain undefined. In our investigation with 7-day-old SPF chickens, we administered an MS-Y bacterial solution (200 µl, 1 × 10^9^ CCU/ml) through eye and nose droplets, collecting spleen samples on days 3, 6, and 12 post-infection. Comprehensive analyses utilizing histopathology, electron microscopy, TUNEL assay, qRT-PCR, and western blot were employed. Results demonstrated that MS-infection downregulated T-SOD, GSH-PX, and CAT, while concurrently elevating iNOS, NO, and MDA levels. Evidently, MS-induced oxidative stress compromised the spleen’s antioxidant defences. Histological examinations pinpointed splenic damage characterized by lymphocyte reduction and increased inflammatory cell infiltration. Ultrastructural observations revealed clear apoptotic markers, including mitochondrial perturbations and nuclear anomalies. Importantly, MS induced significant spleen tissue apoptosis, as supported by TUNEL assay outputs and gene expression profiles associated with apoptosis. Concurrently, we observed upregulated expressions of mRNAs and proteins affiliated with the NF-κB/MAPK signalling cascade (*p* < 0.05). Collectively, our data elucidate that MS infection induces splenic apoptosis and oxidative disturbances, perturbs tissue integrity, and potentiates the NF-κB/MAPK-mediated inflammatory cascade.

## Introduction

*Mycoplasma synoviae* (MS) stands as a dominant extracellular poultry pathogen, inflicting a range of maladies from avian respiratory diseases and infectious synovitis to eggshell apex abnormalities and arthritis. While MS infections rarely culminate in immediate avian death, their escalating economic toll underscores an intensifying threat to poultry enterprises [1]. This amplified virulence stems not just from the pathogen’s intrinsic malevolence but also from the potentiating effects of its cytoadherence, which escalates the release of detrimental factors and cytokines [2,3]. Moreover, MS infections boost the generation of reactive oxygen species (ROS) in vivo, skewing the cellular redox equilibrium and catalysing mitochondrial disturbances. These imbalances invariably lead to compromised immunity and heightened apoptosis [4,5]. The spleen’s cornerstone role in avian innate immunity is firmly established [6], yet the full spectrum of effects stemming from MS-induced oxidative challenges and apoptosis in this vital organ is still a terrain largely uncharted.

The NF-κB/MAPK signalling pathways play a central role in mediating cellular responses to external stimuli, often intertwining with inflammation and oxidative stress [7,8]. Elevated ROS levels have been observed to stimulate IL-1β through ERK1/2 and MAPK phosphorylation, subsequently activating NF-κB [9], a redox-sensitive transcription factor governing numerous inflammatory gene transcriptions [10], such as TNF-α and IL-1β. The TLR4/NF-κB pathway regulates these cytokine secretions, while NF-κB can also activate MAPK, a kinase family crucial for cellular differentiation, proliferation, and apoptosis regulation [11]. The MAPK/ERK pathway has been extensively scrutinized in the context of oxidative stress-induced apoptosis [12,13].

Pathological conditions characterized by oxygen radical surpluses can induce oxidative stress, resulting in mitochondrial dysfunction and apoptosis [14]. There’s a marked rise in oxidative stress indicators, including ROS and malondialdehyde [15], contrasted with diminished antioxidant metrics like superoxide dismutase and glutathione peroxidase [16]. TNF signalling plays a central role in apoptosis modulation. In response to intracellular stress, pro-survival BCL-2 protein inhibition ensues, succeeded by a surge in pro-apoptotic Bax. This alteration augments outer mitochondrial membrane permeability, escalating caspase-3, and −8 gene abundance and subsequently inducing apoptosis. Recent studies highlight accentuates oxidative stress and inflammation as key apoptosis precursors [17]. The exact role of MS in instigating apoptosis in chicken splenocytes, however, remains ambiguous.

The primary aim of this study was to elucidate the impact of MS-infection on chicken spleen. Spleen samples were obtained at various intervals to assess histopathological and ultrastructural alterations. We measured the levels of oxidative stress, inflammation, and apoptosis. Our findings underscored significant ultrastructural damage, compromising the spleen’s structural integrity. Moreover, MS-infection not only fostered oxidative stress and triggered apoptosis but also elevated mRNA and protein expressions of associated inflammatory genes. This investigation offers insights into the mechanisms influenced by MS-infection, paving the way for future therapeutic studies to combat such infections.

## Materials and methods

### Ethics statement

This study strictly adhered to the guidelines set by the Forestry University (protocol approval number PZCASFAFU22015) and complied with the Regulations for the Administration of Affairs Concerning Experimental Animals as mandated by the State Council of the People’s Republic of China.

### MS and culture conditions

The MS-Y strain, previously isolated and characterized by our laboratory [18], was cultured in Modified Frey’s medium. This cultivation occurred at 37°C under an atmosphere containing 5% CO_2_, following the methods we’ve previously described [19]. Notably, the culture exhibited a transition in colour from red to orange during its mid-exponential growth phase. For experimental challenges with chickens, MS with a density of 1 × 10^9^ CCU/ml was used.

### Experimental chickens, infection and sample collection

One-day-old SPF chickens were sourced from Jinan Spafas Poultry Co., Ltd. (Shandong, China) and acclimated for a week under experimental conditions, with unrestricted access to feed and water. The cohort was bifurcated into two distinct groups, each with 15 chickens spread across three replicates: a control and an MS-infected group. To prevent cross-contamination, control birds were maintained in isolation. As previously documented [20], challenges utilized the MS-Y strain (1 × 10^9^ CCU/ml), administered via ocular and nasal pathways. At post-infection days 3, 6, and 12, chickens were euthanized. Subsequent spleen extractions were sectioned for antioxidant, cytokine activity, histopathological, and ultrastructural studies. Residual samples were preserved at −80℃ for extended analyses.

### Histopathological and ultrastructural observation

Spleen samples destined for histopathological analysis were fixed in 10% buffered formalin for 12 hours, then dehydrated through an ethanol gradient, and embedded in paraffin. Four-micrometre sections, once dewaxed, underwent haematoxylin staining for 5 minutes, followed by a 5-minute eosin counterstain. Microscopic evaluations employed an Eclipse Ci-L (Nikon, Japan), with imaging captured via a DS-F12 system (Nikon, Japan).

For ultrastructural assessments, samples first underwent fixation in 2.5% glutaraldehyde, followed by a 15-minute rinse in 0.2 M phosphate buffer (pH 7.2). Post-fixation employed 1% osmium tetroxide for an hour. A subsequent ethanol gradient dehydration preceded embedding in epoxy resin. After drying for 4 hours, a metal film was applied to the 1 mm^3^ spleen tissue sections using ion sputtering equipment. These prepared sections were visualized under a transmission electron microscope (JEOL., Ltd. Japan) and a scanning electron microscope (Hitachi 7650, Tokyo, Japan).

### Assessment of apoptosis using the TUNEL assay (terminal deoxynucleotidyl transferase-mediated dUTP nick end-labeling)

The TUNEL assay was utilized to identify apoptotic cells in chicken spleen. Tissues were fixed in 10% formalin, dehydrated, embedded in paraffin, and sectioned onto glass slides. Following nuclear labelling with diaminobenzidine and horseradish peroxidase, apoptotic cells were visualized using a confocal microscope (Eclipse Ti2, Nikon, Japan) in light-protected conditions.

### Determination of oxidative stress-related parameters

Spleen tissue was harvested and homogenized in saline to produce a tissue suspension. After centrifugation at 3,000 g for 10 minutes at 4℃, the supernatant enriched in proteins was collected. Using kits according to the manufacturer’s instructions, we determined the levels of Malonaldehyde (MDA, Cat no. A003–1), Nitric Oxide (NO, Cat no. A012), Catalase (CAT, Cat no. A007–1), Inducible Nitric Oxide Synthase (iNOS, Cat no. A014–1), Superoxide Dismutase (T-SOD, Cat no. A001–1), and Glutathione Peroxidase (GSH-Px, Cat no. A006). The absorbances were measured at 532 nm, 530 nm, 405 nm, 530 nm, 450 nm, and 412 nm, respectively. All the kits were obtained from Nanjing Jiancheng Bioengineering Institute, Nanjing, China.

### RNA isolation and qRT-PCR

Total mRNA of spleen tissue was extracted by the Trizol method, and the concentration and purity of mRNA were determined by NanoDrop (Thermo, USA). Then 1 μg mRNA was reverse transcribed into cDNA. Subsequently, relative mRNA abundance was quantified using SYBR Green (Monad, Wuhan, China) on a Step One Plus machine (ThermoFisher, USA). The fold changes of gene expression were calculated by the 2^−ΔΔCt^ method [21]. The primer sequences are shown in Table 1.

**Table 1.** Primer sequences of the genes for qRT-PCR.

Genes	Primers(from 5’to 3’)	Primers origin	Bp
NF-KB	F:GTGTGAAGAAACGGGAACTG R:GGCACGGTTGTCATAGATGG	NM 205129	205
JNK	F:TGACCGAGTGAGGAGACGAT R:ACTGTATCGAACGCAGCACA	NM 205095.1	211
ERK	F:AGAATCTCACAGCGTCTCGC R:GGTGTGATTCATCAGCATCTTCA	NM 205388.1	235
TLR4	F:CATACAAGCCACTCCAAGCC R:AGGATTTCCAGGGCTGAGTC	NM 001030693.1	96
p65	F-CAGCCCATCTATGACAACCG R-CAGCCCAGAAACGAACCTC	D13721151	151
IkBα	F:GGCAGATGTGAACAAGGTGA R:TATCTGCAGGTCAGCTGTGG	NM 001001472.2	118
iNOS	F:CCCTCCAGCTGATCAGACTATC R:GTGTGCAAGCCGGAATCTTTT	NM 204961.1	86
TNF-a	F:CAGATGGGAAGGGAATGAAC R:AGAACAGCACTACGGGTTGC	JN942589.1	238
I-1β	F:AGCAGCCTCAGCGAAGAGACC R:GTCCACTGTGGTGTGCTCAGAATC	NM 204524.1	137
II-6	F:GCTCGCCGGCTTCGA R:GGTAGGTCTGAAAGGCGAACAG	AJ309540	254
Bax	F:GGGGTACGTCAATGTGGTCA	XM 015274882.1	58
	R:AGGAAGGCGGTGGGATAATG		
Caspase-3	F:TACTCCTGGAGGAACGCAGC R:TGCCACTCTGCGATTTACACG	NM 204725.1	123
Caspase-8	F:F:CCCTGAAGACAGTGCCATTT R:GGGTCGGCTGGTCATTTTAT	NM 204592.2	106
P53	F:GCTGAACCCCGACAATGAGA R:TTTGCAGCAGTTTCTTCCCG	NM 205264.1	145
Bcl-2	F:GATGACCGAGTACCTGAACC R:CAGGAGAAATCGAACAAAGGC	NM 205339.2	150
β-actin	F:GAGAAATTGTGCGTGACATCA R:CCTGAACCTCTCATTGCCA	JN639846.1	107

### Western blot analysis

Western blot was used to measure the related proteins. Proteins were extracted from spleen samples, as delineated in a previous study [22]. After SDS-PAGE separation and transfer to nitrocellulose membranes, the proteins were blocked and incubated with primary antibodies from Beijing Bioss Biotechnology. Following a wash, secondary antibodies were applied. Protein bands were visualized using Beyotime’s ECL reagent and quantified with Image J.

### Statistical analysis

Experiments were conducted in triplicate (*n* = 3). Data analysis was carried out using SPSS (version 24.0), with significance between groups determined by t-test. Data are presented as mean ± SD, with *p* < 0.05 indicating statistical significance.

## Results

### Histopathological and ultrastructural changes in chicken spleens

**Figure 1.**
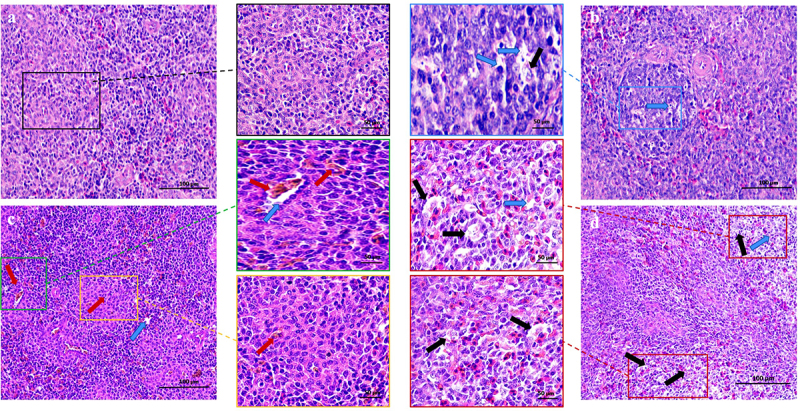
Histopathological changes of chicken spleen tissues (200×). (*n* = 3). Experimental groups including (a) control group (b) MS-infection 3 dpi group (c) MS-infection 6 dpi group (d) MS-infection 12 dpi group. Black arrows represent spleen tissue losing its compact arrangement with increased infiltration of inflammatory cells. Green arrows represent lymphocyte infiltration. Red arrows represent necrotic fragments of lymphocytes. Yellow arrows represent bleeding.

Upon dissection, MS-infected spleen tissue appeared noticeably smaller and lighter than normal spleen tissue. To evaluate MS’s damaging effect on chicken spleen, we utilized H&E staining. Figure 1 illustrates that in the control group, lymphocytes were orderly arranged, with distinct delineation between white and red medulla, and the central artery displayed typical morphology (Figure 1(a), black box). Conversely, the MS-infected spleen showcased hallmark signs of injury: pronounced inflammatory cell infiltration with numerous vacuolated cells (Figure 1(b), blue arrows), diminished and disordered lymphocytes (Figure 1(c), yellow arrows), and the presence of minimal iron-rich haemoglobin (Figure 1(c), red arrows). Notably, necrotic features like nuclear fragmentation and nucleolysis were evident (Figure 1(d), black arrowheads).

**Figure 2.**
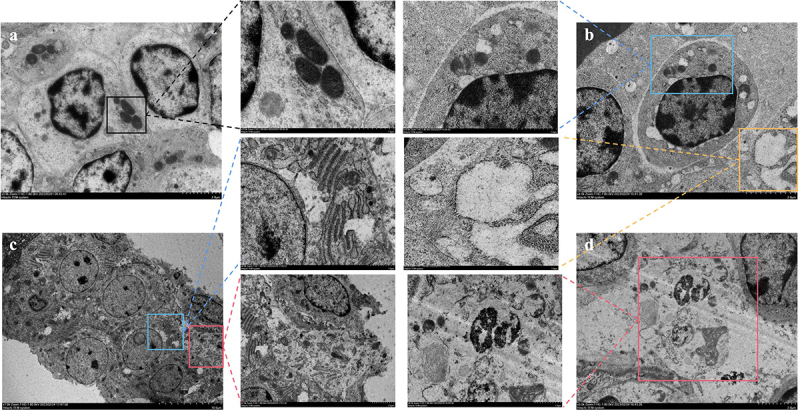
Shows the effect of MS-infection on ultrastructural changes in the spleen of chickens (×6,000). (a) control group, the normal mitochondrial structure (black box) (b) MS-infection 3 dpi group, with mitochondrial swelling, cristae disorders, and vesicles (blue box); (c) MS-infection 6 dpi group with extensive formation of vesicles (yellow box); (d) MS-infection 12 dpi group, presence of apoptotic cells with nuclear lysis, cell membrane rupture, and cell necrosis (red box). (*n* = 3).

Further, we examined ultrastructural modifications in spleen cells. As depicted in Figure 2, the control group displayed no notable ultrastructural alterations, maintaining an intact cell membrane and abundant, intact mitochondria (Figure 2(a)). In stark contrast, the MS-infected group presented classic apoptotic markers such as mitochondrial swelling, cristae disruption, and vacuolisation (Figure 2(b), blue box), complemented by extensive vesicle formation (Figure 2(b/c), yellow box), cell membrane rupture, cytoplasmic spillage (Figure 2(c), red box), and nuclear lysis (Figure 2(d), red box). Collectively, these findings substantiate that MS-infection induces necrosis and inflammation in the chicken spleen.

### Expression of apoptotic genes and TUNEL assay in chicken spleens

Recent literature underscores the intrinsic link between tissue pathology and levels of apoptosis [23]. Building on this foundation, our initial step involved TUNEL staining of spleen sections from the 12d/dpi group (refer to Figure 3). The findings were telling: the spleen tissue from MS-infected chickens exhibited a pronounced increase in apoptosis, as evident from the surge in positively stained nuclei compared to the control group.

**Figure 3.**
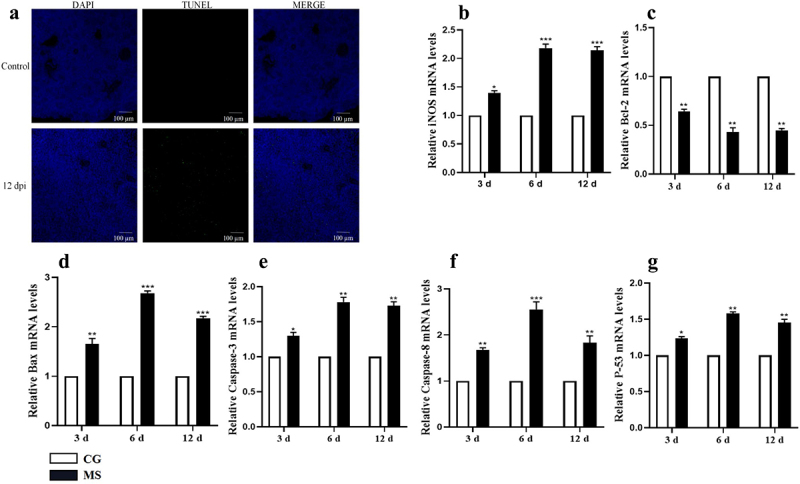
(Continued)

Venturing further, we probed the mRNA expression profiles of pivotal apoptosis-related genes, namely iNOS, caspase-3, caspase-8, Bax, p53, and Bcl2, using qPCR techniques. Figure 3 elucidates the impact of MS-infection: a significant enhancement in the mRNA expression of cysteinyl asparaginase 3 was observed (*p* < 0.05). Concurrently, genes associated with the mitochondrial apoptosis pathway, notably bax and p53, exhibited noticeable upregulation. In stark contrast, the expression of the anti-apoptotic gene, Bcl-2, diminished notably post-infection (*p* < 0.05). Corroborating these findings, the TUNEL assay unequivocally demonstrated the induction of apoptosis in chicken splenocytes due to MS-infection. Taken together, our data robustly accentuates the pronounced pro-apoptotic consequences of MS-infection on spleen tissues.

**Figure 3.**
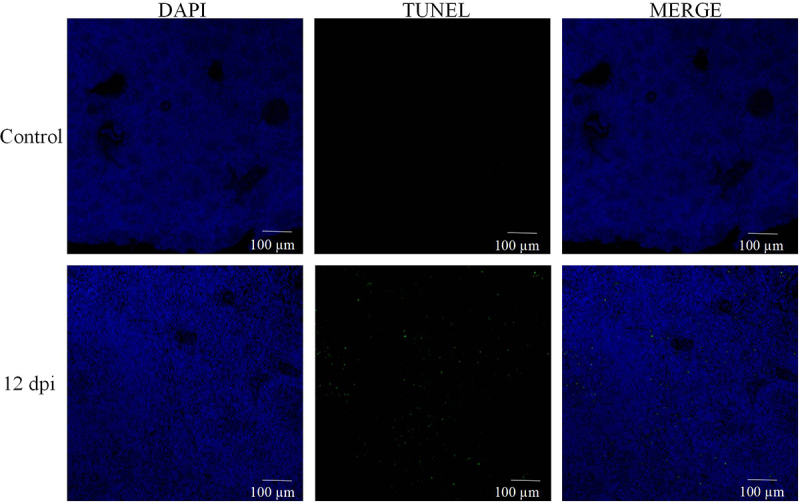
MS induces apoptosis in the spleen tissue of carp. (a) TUNEL staining of spleen tissue; (b-g) apoptosis-related gene iNOS, Bcl-2, Bax, caspase-3, caspase-8, caspase-9, and *p*-53 mRNA expression levels; n=3 for each group; *p< 0.05, ***p< 0.001 versus control.

### Impact of MS-infection on antioxidative functions in spleen tissues.

**Figure 4.**
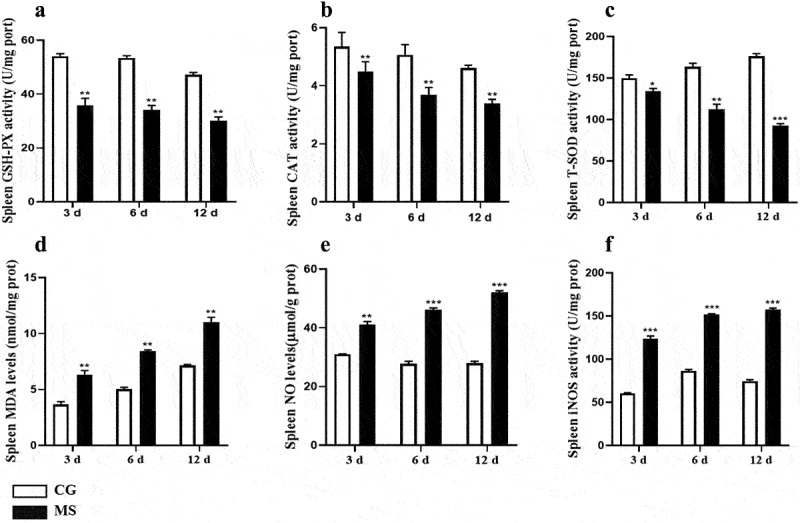
Shows the effect of MS-infection on cytokine mRNA. (a) western bolt strip of TNF-α‘IL-6‘IL-1 ‘and β-actin (*n* = 3). (b-d) the ratio of relative protein levels to β-actin. (e-g) TNF-α‘IL-6 and IL-1 βmRNA expression level. Different small letters indicate statistical significance (*p* < 0.05) between the two experimental groups at the same time point.

Apoptosis initiation is often linked to oxidative stress [24]. In our investigation, we quantified the activities of GSH-PX, CAT, T-SOD, and iNOS, along with the concentrations of NO and MDA (Figure 4). MS-infection consistently reduced the activities of antioxidant enzymes T-SOD, CAT, and GSH-Px across all examined time points when compared to the control Figure 3(a-c). In contrast, the activities of iNOS, NO, and MDA increased for each of these intervals Figure 3(d-f). Notably, the alterations in antioxidant indicators due to MS-infection exhibited a time-dependent pattern.

### Inflammatory response and NF-κB pathway activation in chicken spleens

**Figure 5.**
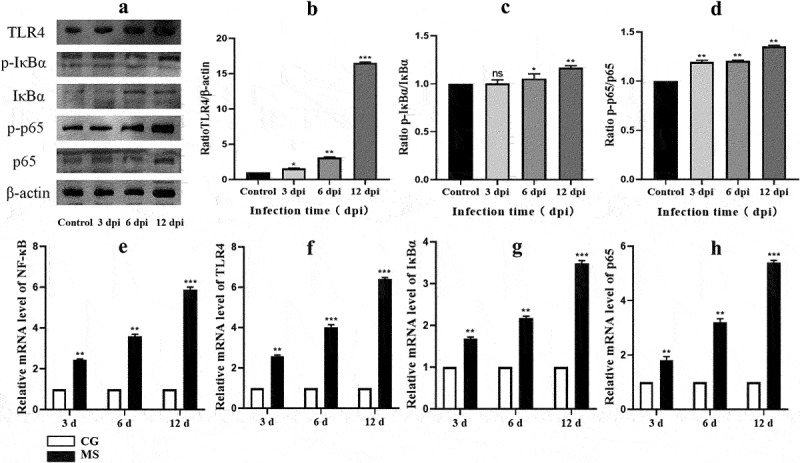
The activation of the NF-κB pathway. (a) western bolt strip of NF-κB related proteins TLR4, IκBα, p-IκBα, p65, p-p65,TLR4 and β-actin (*n* = 3); (b-d) the ratio of relative protein levels of TLR4/β-actin, p-IκBα/IκBα, p-p65/p65. (e-h) NF-κB, TLR4, IκBα, p65 mRNA expression levels. Different small letters indicate statistical significance (*p* < 0.05) between the two experimental groups at the same time point.

The activation of the NF-κB signalling pathway is often characterized by the phosphorylation of p65. In our current findings, represented in Figure 5, we observed a notable surge in both mRNA and protein levels of pivotal markers such as p-IκBα/IκBα, p-p65/p65, and TLR4 within chicken spleen tissues. This upregulation was particularly significant at the time points of 6 dpi and 12 dpi, with p-values indicating statistical significance (*p* < 0.05 or *p* < 0.01). Such observations highlight the potential activation of the NF-κB pathway during these stages post-infection Figure 4.

**Figure 6.**
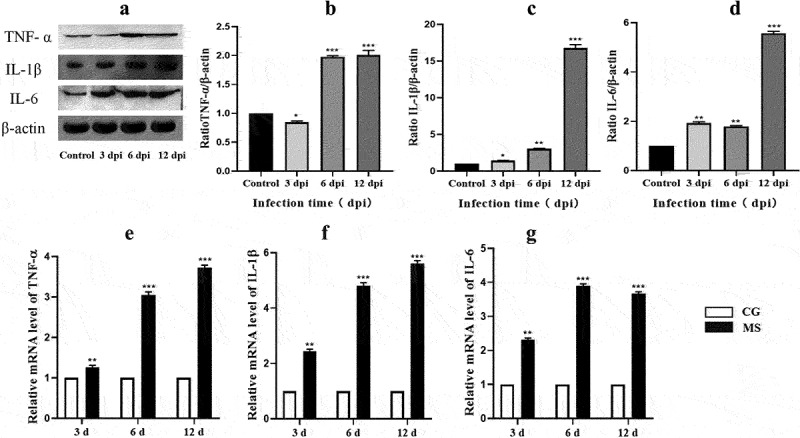
Shows the effect of MS-infection on oxidative stress-related parameters measured on 3, 6 and 12 day in chicken spleen. The assessed parameters are: (a) GSH-PX activity, (b) CAT activity, (c) T-SOD activity, (d) MDA content, (e) NO content and (f) iNOS content. All the bar graphs show mean results ± SD (*n* = 3). Experimental groups are represented as control group and MS-infection group. Different small letters indicate statistical significance (*p* < 0.05) between the two experimental groups at the same time point.

Given the pivotal role the NF-κB signalling pathway plays in regulating inflammatory responses [25], our study delved deeper into its downstream impacts. As delineated in Figure 6, there was a pronounced upregulation in the protein expression of pro-inflammatory cytokines: TNF-α, IL-6, and IL-1β within chicken spleens following MS infection, reaching significant thresholds (*p* < 0.05 or *p* < 0.01). Additionally, our analysis uncovered a congruent increase in mRNA levels of these central inflammation-associated cytokines associated with the NF-κB pathway. This trend was closely tied to the duration of MS-infection. Summarily, MS amplifies inflammatory responses by both curtailing the expression and activity of antioxidant enzymes and simultaneously triggering the NF-κB pathway.

### The activation of the MAPK pathway

**Figure 7.**
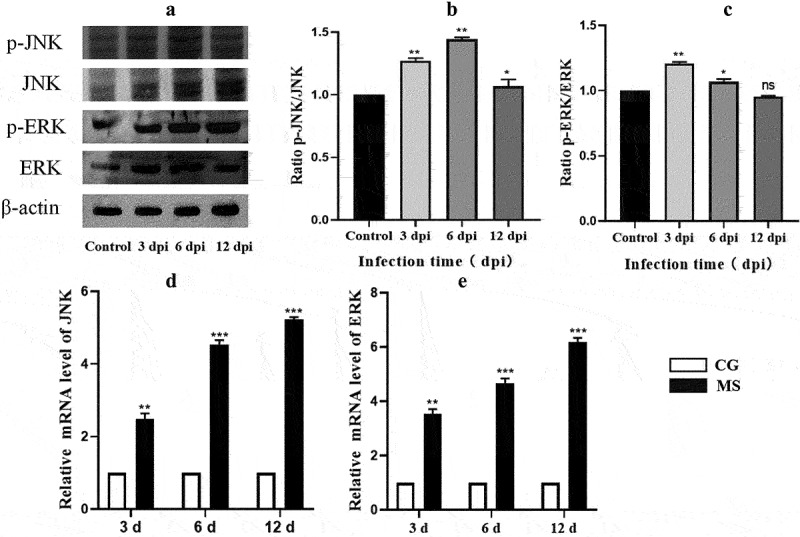
The activation of the MAPK pathway. (a) phosphorylated protein expressions of JNK, ERK in chicken spleens. (*n* = 3). (b,c) the ratio of relative protein levels of p-JNK/JNK, p-ERK/ERK. (d,e) JNK‘ERK mRNA expression level. Different small letters indicate statistical significance (p <0.05) between the two experimental groups at the same time point.

The MAPK pathway is an important signal pathway which could regulate various pathological mechanisms, including oxidative stress, dysfunction, and inflammation. To assess the relationship between apoptosis and the MAPK pathway, we examined the expression of JNK and ERK in chicken spleens. As is presented in Figure 7, the expression of both mRNA and phosphorylated proteins of JNK and ERK were elevated (*p* < 0.05 or *p* < 0.01). Compared with the control group, the expression of MAPK pathway became higher and higher with the prolongation of MS-infection. This indicated that MS-infection could activate the MAPK pathway.

## Discussion

*Mycoplasma synoviae* (MS) is a primary avian pathogen, known for causing respiratory maladies and synovitis in chickens. Once the host falls prey, MS promotes the release of immune modulators, maintaining a chronic inflammatory backdrop [26]. Previous studies have shown MS’s penchant for infecting a diverse spectrum of non-phagocytic cells from fibroblasts and HeLa cells to chicken red blood cells (RBCs) [27]. Its sinister reach extends to extrapulmonary tissues such as the heart, blood, liver, brain, and notably, the spleen, adeptly dodging the host’s innate immune surveillance [27–29]. Compounding its virulence, MS’s adept cell-adhesion attributes usher in a slew of virulence factors, inclusive of superoxide radicals, culminating in immune destabilization [30]. Yet, the precise mechanisms underlying MS’s chronic virulence in chicken infections are still not fully understood.

In the scientific annals, there’s an accumulating consensus linking mycoplasma infections to inflammation, oxidative tumult, and ensuing apoptosis [31,32]. A suite of studies spotlighted how MS not only modulates a plethora of immune genes in chicken macrophages but also impacts apoptosis genes within chicken chondrocytes [30,33]. Enter the spleen, the body’s paramount peripheral immune bastion, teeming with lymphocytes (LYM) and macrophages, which are indispensable for immune orchestration [34]. Ergo, it stands to reason that any foreign pathogenic assault on the spleen will imperil the host’s overarching immune defences. Lending credence to this, investigations have underscored how mycoplasma onslaughts induce oxidative upheavals, thereby compromising mitochondrial integrity and wreaking DNA havoc in lymphocytes [35,36], potentially precipitating lymphocytic damage and systemic immune dysfunction.

Delving deeper into the MS-inflicted carnage, our current exploration meticulously probed the repercussions of MS-infection on the chicken spleen. The triumvirate of SOD, CAT, and GSH, revered for their endogenous free radical-quenching prowess, are pivotal in preserving the body’s oxidative equilibrium [37]. Their attenuated activities, as unveiled in our observations, serve as a distress beacon, signalling the antioxidant system’s siege by rampaging free radicals. Our meticulous assessments, gauging the oxidant-antioxidant continuum post-MS-infection in chicken spleens, unmasked an overproduction of MDA and a startling nosedive in the antioxidant vanguards T-SOD, CAT, and GSH-Px (*p* < 0.05). This disarray alludes to an ROS deluge in the spleen, catalysing heightened oxidative stress an assertion further buttressed by our temporal data mapping oxidative stress dynamics post-infection (*p* < 0.05). Consistent with previous studies [38–40], our histopathological examinations evaluations showed clear changes in spleen structure in chickens affected by MS, accompanied by an upsurge in inflammatory cytokines.

Reactive Oxygen Species (ROS), primarily originating from mitochondria, are not just markers of oxidative damage but also play a key role as mediators of apoptosis [41,42]. Ultrastructural microscopy revealed evident signs of apoptosis, such as fragmented mitochondrial cristae and significant mitochondrial swelling. Additionally, our TUNEL assay showed heightened nuclear staining in chicken spleen cells after MS-infection, suggesting intensified apoptosis. Analysis of gene expression, encompassing iNOS, Bax, Caspases-3, Caspases-8, Bcl2, and p53, demonstrated that MS-infection was promoting apoptosis in the chicken spleen. Importantly, we detected an overexpression of p53, an increase in Bax, and a reduction in Bcl-2, all pointing to an activated mitochondrial apoptosis pathway [43–45].

While ROS indisputably wreak oxidative havoc, their nuanced role as molecular beacons triggering inflammation and immune cytokine genesis can’t be overlooked. The probable entanglement of ROS in myriad inflammatory ailments has been posited, with cytokines serving as bellwethers of inflammation [46, 47]. To decipher the MS modus operandi in inducing splenic inflammation, our lens zoomed into the NF-κB/MAPK signalling axis, a renowned mediator of cellular reactions to exogenous stimuli, with strong ties to inflammation and oxidative stress. Our data, in harmony with prior findings (Figure 5), accentuated the NF-κB pathway’s centrality in inflammation, highlighting the NF-κB/MAPK nexus in the MS infectious saga. Given the MAPK pathway’s evolutionary conservation and its multifaceted role spanning proliferation to apoptosis [48,49], our detailed examination revealed the pathway’s activation during MS’s infectious onslaught. We thereby posit a hypothesis: MS masterminds oxidative and inflammatory turmoil in the chicken spleen via the NF-κB/MAPK conduit.

## Conclusion

In conclusion, the MS-inflicted assault on chickens manifests predominantly in the spleen, with stark disruptions in its structural integrity and discernable morphological aberrations. Our in-depth examination revealed that such deleterious effects stem from a concerted interplay of mitochondrial pathway-driven oxidative stress and apoptosis, complemented by the machinations of the NF-κB/MAPK pathway, precipitating inflammation. This cascade of events culminates in a profound disturbance of the immune equilibrium within the host. Collectively, this investigation not only unveils the intricacies of immune compromise in chickens following MS infection but also paves the way for subsequent research endeavours aimed at unravelling the nuanced molecular underpinnings dictating immune dysregulation.

## Supplementary Material

Figure 3, A.jpg

## Data Availability

All datasets generated for this study are included in the article/Supplementary Material.

## Highlights

**Highlights** for the manuscript entitled “*Mycoplasma synoviae* induce spleen tissue damage and inflammatory response of chicken through oxidative stress and apoptosis”

- *Mycoplasma synoviae-*induced pathological changes in spleen.
- MS causes disruption of the antioxidant system in the spleen tissue of chicken.
- MS triggers apoptosis of the mitochondrial pathway in chicken spleen tissue.
- MS mediates oxidative stress, apoptosis and inflammatory response in chicken spleen tissue through activation of the NF-κB/MAPK pathway.

Prof. Yijian Wu on behalf of the co-authors
