# Supplementary figures and images for: Mycoplasma synoviae induce spleen tissue damage and inflammatory response of chicken through oxidative stress and apoptosis
Source: Virulence. 2025 Sep 16;15(1):2283895. doi: 10.1080/21505594.2023.2283895 (PMC12506914; doi:10.1080/21505594.2023.2283895)

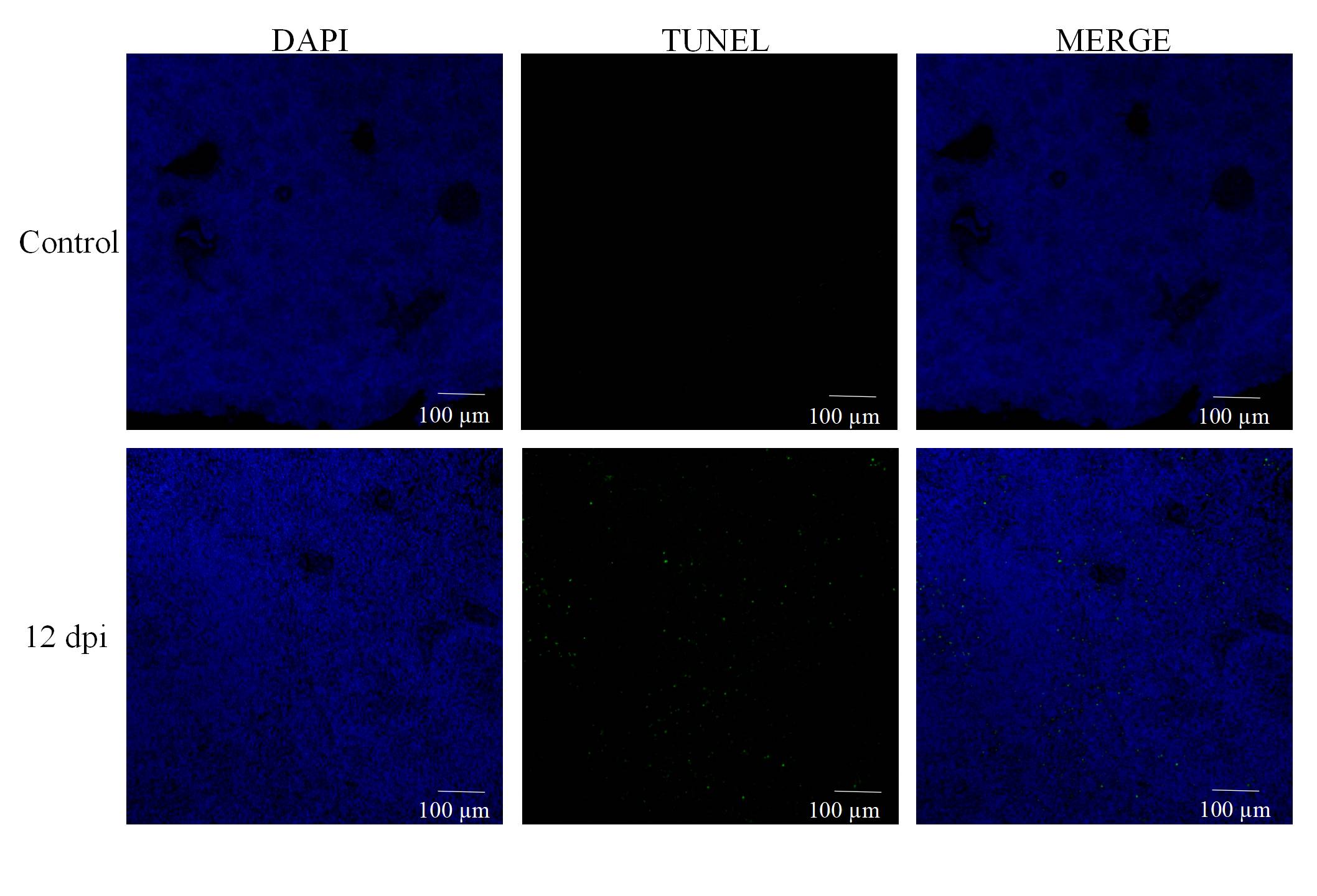

Supplement: Figure 3, A.jpg [file KVIR_A_2283895_SM0375.jpg]
